# Application of Red and Blue LED Light on Cultivation and Postharvest of Tomatoes (*Solanum lycopersicum* L.)

**DOI:** 10.1155/2024/3815651

**Published:** 2024-09-03

**Authors:** Abdullah Bin Arif, Agus Budiyanto, Setiawan, Tri Cahyono, Tri Ratna Sulistiyani, Tri Marwati, Siti Mariana Widayanti, Setyadjit, Lamhot Parulian Manalu, Himawan Adinegoro, Nenie Yustiningsih, Mulyana Hadipernata, Irpan Badrul Jamal, Indra Budi Susetyo, Heny Herawati, Kasma Iswari, Risfaheri

**Affiliations:** ^1^ Research Center for Agroindustry National Research and Innovation Agency, Central Jakarta, Indonesia; ^2^ Research Center for Horticulture National Research and Innovation Agency, Central Jakarta, Indonesia; ^3^ Research Center for Sustainable Production Systems and Life Cycle Assessment National Research and Innovation Agency, Central Jakarta, Indonesia; ^4^ Research Center for Biosystematics and Evolution National Research and Innovation Agency, Central Jakarta, Indonesia; ^5^ Research Center for Food Technology and Processing National Research and Innovation Agency, Central Jakarta, Indonesia

## Abstract

Currently, light-emitting diode (LED) technology has produced a more energy-efficient and versatile technology as an artificial lighting system that can be applied in the agricultural sector. Artificial lighting technology has been proven to be effective in increasing the production of agricultural products, especially horticultural commodities. As one of the primary horticulture commodities, tomatoes are the most common crop produced in controlled environments with LED artificial lighting. The focus of this study is to describe the application of LED lights in tomato cultivation and postharvest. We provide an amalgamation of the recent research achievements on the impact of LED lighting on photosynthesis, vegetative growth, flowering, production, and postharvest of tomatoes. Red-blue (RB) lighting induces photosynthesis; increases the content of chlorophyll a, chlorophyll b, and carotenoids in tomato leaves; regulates vegetative growth in tomatoes; and increases the production of tomatoes. In postharvest tomatoes, blue LED lighting treatment can slowly change the color of the tomato skin to red, maintain hardness, and increase shelf life. Future research may be carried out on the effect of LED artificial lighting on tomatoes' phytochemical, antioxidant and other crucial nutritional content. Different LED wavelengths can be explored to enhance various bioactive compounds and health-promoting components.

## 1. Introduction

Recently, agricultural research, especially using light-emitting diodes (LEDs), has produced a more energy-efficient and versatile technology as a lighting system [1–5]. Light-emitting diode (LED) illumination is widely used in cultivating several types of plants, especially horticultural plants because its power consumption is lower and its light efficiency is higher than conventional fluorescent lights [6]. LEDs can also be used in various horticultural lighting applications, such as tissue culture, controlled environment research, and supplemental and photoperiod lighting for greenhouses [2]. The application of LEDs in agricultural cultivation increases yield, quality [1, 7], photosynthesis efficiency [8–10], and the content of secondary metabolite compounds [11–13] in agricultural commodities, especially horticultural commodities. However, the effect of LEDs on plant growth and quality is species-specific, and the effect is influenced by cultivation conditions [6].

Besides influencing the cultivation system, Bantis et al. [7] reported that lighting treatment during storage affects the shelf life and quality of horticultural products, which has the potential to (1) increase soluble carbohydrates, which are substrates for respiration during storage; (2) increase or maintain levels of vitamin C, anthocyanins, total phenolics, sugars dissolved, and antioxidants; and (3) enhance or maintain visual appearance by the accumulation of pigments (lycopene, carotenoids, and anthocyanins). Light-emitting diodes' (LEDs) illumination has the potential to play an important role in postharvest horticultural products [14]. Perera et al. [15] also reported that applying light-emitting diodes (LEDs) in postharvest horticultural commodities is a new, nonchemical, and residue-free technique to preserve safe and nutritious fresh horticultural crop commodities. LED illumination during storage can be an alternative solution to reduce losses during the postharvest process and maintain the quality of horticultural products. This is because the application of LED can extend shelf life and produce low heat emissions, which allows its use in cold storage rooms and refrigerators as well as in refrigerated container cars, where the light spectrum can be adjusted along with other positive features of LED lights. Therefore, the potential application of LED utilization in horticultural products continues to grow, especially in tomato commodities.

As one of the most widely consumed vegetables in the human diet, tomatoes are a source of minerals, vitamins, and antioxidant compounds that benefit human health [16, 17]. Therefore, innovative cultivation technology is needed to increase tomato production and its nutritional content. In addition, tomatoes are one of the horticultural commodities that have a high weight loss during storage, and the shelf life of tomatoes is still relatively short. Postharvest loss can be caused by several factors, including physiological, pathological, physical, and a combination of these three factors. Ethylene production, chlorophyll degradation, lycopene synthesis, and cell wall softening are the main factors of tomato ripening. The color and texture of fresh tomatoes are the main quality attributes directly related to marketing and consumer acceptance. Apart from being a determinant of color, lycopene and carotenoids are also beneficial to health [18]. The potential use of LEDs in tomato cultivation and postharvest systems needs to be discussed and studied in more depth. The focus of this review is to describe the application of LED to tomato plants in the field (cultivation) and during the postharvest phase.

## 2. Impact of LED on the Growth and Development of Tomato

Light, water, and oxygen are the critical factors significantly influencing plant growth and development. Light is essential in establishing desired morphological and photosynthetic characteristics among these factors. Light is an important factor in the photosynthesis process for food preparation to continue life processes and also controls many developments and physiological responses throughout the plant life cycle [19, 20]. The spectrum greatly influences plant growth and development response to light intensity and quality control [21]. Plants need sunlight as a source of sufficient natural light to synthesize photosynthesis [22]. However, the availability of natural light from sunlight used by plants for photosynthesis is influenced by several factors, including the influence of clouds, rain, and other climatic factors. Several studies have discussed the positive influence of artificial lighting on plant growth and the development of LED lighting. This effect is influenced by the spectral composition of the LED source, lighting duration, and plant species [23]. Light-emitting diodes (LEDs) have been proven to be low-cost light sources and emit minimal heat [24]. LEDs produce adjustable light intensity and suitable wavelengths, making them suitable for various plant species. LEDs also provide the advantage of reducing oxidative stress due to excessive light energy. The wide variety of LED colors allows precise wavelength control without additional filters [25]. A lighting system using specific wavelengths can enrich the nutritional content of plants; however, special attention should be paid to the stress that artificial light may exert on photosynthetic mechanisms and its consequences on biomass accumulation [23].

Secondary metabolites show different characteristics than primary metabolites, involving carbohydrates and amino acids biosynthesized by plants. Phenolics and flavonoids, which are included in the phenolic compounds, are secondary metabolite compounds produced by plants and play a role in plant adaptation to changes in the biotic and abiotic environments [26, 27]. As blue and red pigments, phenolic compounds function as antioxidants and protect against ultraviolet light. Anthocyanins are related to the color of flowers and fruit and function as insect attractants and antimicrobials. Blue light increases anthocyanin synthesis by increasing the expression of anthocyanin synthase and chalcone synthase (CHS) genes, and the anthocyanin protects cells from high-light damage. Carotenoids are terpenoid compounds that function as accessory pigments of orange and yellow lights [28]. Carotenoids can limit damage to membranes caused by excess light because they play a role in the absorption of free radicals, as well as being able to absorb light energy, which is in the spectral region where chlorophyll does not exploit enough, and then transfer it to chlorophyll, thereby increasing the efficiency of plant photosynthesis [28]. The defence mechanism of carotenoids and chlorophyll in excess light is nonphotochemical quenching (NPQ), where carotenoids are directly involved in the dissipation of excess excitation light as heat occurs in the outer antenna of photosystems II. All mechanisms to remove this trapped energy before passing it on to the electron transport chain are called NPQ. There are two different mechanisms depending on pH or energy: the first involves zeaxanthin (Zea) (quenching type 1) and the second involves the carotenoid lutein (Lut) (quenching type 2). The primary carotenoid in tomatoes is lycopene, which causes the red color of tomatoes. In pharmacology, lycopene has been reported to have anticancer, anti-inflammatory, antidiabetic, antiallergic, antiatherogenic, antithrombotic, antimicrobial, antioxidant, vasodilator, and cardioprotective activities [16]. In addition, lutein and *β*-carotene are also important carotenoids taking part in the light-harvesting complex in leaves.

### 2.1. Application of LED Lighting in Tomato Cultivation

#### 2.1.1. Photosynthesis

Plant growth and development are influenced by light, and the response depends on the species and cultivar [29]. Photosynthesis is an essential plant reaction that converts energy (light) into chemical energy stored in organic compounds. The high light intensity can cause a lower accumulation of photosynthetic pigments [30]. Generally, photosynthesis is impaired in plants growing under monochromatic light [31–33]. In addition, plants growing under monochromatic light experience impaired photomorphogenesis due to the unbalanced activation of photoreceptors that mediate light-dependent plant development [34]. In tomato plants, photosynthesis in seedlings under red light was inhibited by stomatal closure and caused a reduction in CO_2_ assimilation [35]. The tomato plantlets showed at least a threefold decrease in photosynthesis rate and a significant abnormal stem elongation when grown under 100% red light [36]. Furthermore, tomato plants grown under far-red light reduced leaf's maximum photosynthesis, leaf mass, thickness, and nitrogen and increased the resistance to CO_2_ diffusion [37]. Plants grown under monochromatic red light show lower maximum photochemical efficiency, unresponsive stomata, and lower photosynthetic capacity than plants grown with supplemental blue light [21, 38].

Meanwhile, blue light in tomato cultivation increases photosynthesis, which helps provide a nitrogen source and stimulate nitrate metabolism [39, 40]. Blue light plays a role in regulating growth because it is involved in several critical plant responses such as phototropism, photomorphogenesis, stomata opening, chloroplast development, and leaf expansion [41–43]. In supplemental lighting indoors and in greenhouses, blue light has less or no growth inhibitory effect, so a small amount of blue is always included in the light spectrum [38].

The previous research results reported that a combination of red and blue light has proven effective in encouraging tomato photosynthesis. Red-blue light increased the photosynthetic capacity of photosynthate production in tomato leaves [44]. Photosynthesis of tomato plantlets was efficiently enhanced by increasing the light fraction B in combination with RB light [36]. Red-blue LED increased shoots' fresh and dry weight and leaf area, displaying significantly higher photosynthesis rates [9]. The combination of red and blue light has the most effective photosynthetic waveband. RB light's influence on the photosynthetic pigment concentration increased the total chlorophyll [35]. Plants grown under RB light have the most excellent photosynthetic efficiency because this wavelength range closely coincides with the Chl absorption peak [45, 46]. Tomato plants show higher photosynthesis rates, pigment content, and reduced stomata closure [38]. In contrast, combined red-blue LED light supplementation has significantly increased tomato rubisco activity [47]. The combination of red and blue light speeds up photosynthesis compared to red or blue light alone [48]. Red and blue lights maximally induce photosynthesis, while their combination provides the highest photon efficiency compared to other LED combinations [49]. The proper ratio of red and blue LEDs increases the photosynthesis rate, photosynthetic pigment content, and photosynthetic efficiency in tomato seedlings [50, 51]. The red-blue light spectrum is absorbed by plant leaves to carry out photosynthesis and photosynthesis is maximally induced by blue and red light [52, 53]. The combination of red and blue lights promotes stomata opening and increases CO_2_ uptake and assimilation by leaves [53]. In addition, the red-blue LED lights increase the content of chlorophyll a, chlorophyll b, and carotenoids in tomato leaves [54].

#### 2.1.2. Plant Vegetative Growth

Red and blue light is essential in many plant growth and development factors. As a significant horticultural crop, tomato is often used as a model crop to study plant responses to red and blue light [55–57]. Shoot elongation is one of the most essential morphological characteristics to determine the response of plants to red and blue lights. Blue light inhibits elongation growth by activating cryptochromes, positively correlated with blue light-dependent phosphorylation [58, 59]. In tomato plants, monochromatic blue light is induced in seedlings with the highest rubisco amount, more compact size, and reduced biomass [33]. In addition, an increase in the blue light percentage resulted in a decreased plant height [60]. Increased blue light negatively impacts plant elongation and leaf area, inhibiting cell division and expansion [61–63]. However, the treatment of red LED increased the plant height and stem diameter of tomatoes [64]. In addition, the red pure light induced hypocotyl elongation, cotyledon expansion, plant height, and leaf area [33]. Plants grown under red light show increased hypocotyl elongation, internode spacing, and leaf surface [33]. The leaf area of plants grown under red light is comparable to that of plants grown under red-blue light, but the dry weight is lower due to the reduced leaf mass per area (LMA) on the leaves of plants grown under red light compared to blue and red-blue [33]. Likewise, with the effect of far-red light on tomatoes, Hao et al. [65] reported that far-red light increased stem length and carotenoid content. Far-red light also increased stem length, leaf area, and total plant biomass [66, 67]. Adding FR light increases tomato plants' total dry mass at the vegetative growth stage [68]. In addition, Gou et al. [69] also reported that adding far-red light with LEDs induced stem elongation and plant height, which resulted in a more light interception and increased plant growth. Similarly, Zhang et al. [66] reported higher total plant dry mass under FR light. Far-red light increases the height of tomato plants and shows the expression of shade avoidance syndrome (SAS) [70].

Although high doses of blue light inhibit plant growth and biomass production because the energy is not fully used in photosynthesis, a low percentage of blue light is needed to complement red light for optimal plant growth [71–73]. The right combination of red and blue LEDs can promote photosynthesis and regulate vegetative growth in most species. In tomatoes, 95% red +5% blue light increased the number of nodes (stem segments) [74]. The RB LED improved the total plant biomass of tomatoes [75] and increased the plant height and stem diameter of tomatoes [64]. In addition, the higher temperature combined with RB light is an indisputable optimal regime for tomato growth [76] and routine content in leaves, increased content of young leaf flavonoids, and decreased content of flavonoids in mature leaves [77].

#### 2.1.3. Flowering

As an energy-saving additional lighting option, LED increases plant flowering and photosynthetic efficiency in greenhouse [78, 79]. Light is an essential factor that influences plant growth, including flowering. Xie et al. [80] reported that blue light promoted early tomato flowering. Strawberries under blue light accelerate flowering than red light [81, 82]. In addition, increasing blue light induces faster flowering [83–85]. Blue and red light induce photomorphogenic responses in plants through cryptochromes and phytochromes [86, 87], where light interacts through photoreceptors with flowering genes to regulate flowering [88]. The cry1 and cry2, as cryptochrome receptors, induce flowering by responding to wavelengths of 390–480 nm [89]. Phytochromes, absorbing red and far-red light (700–800 nm), express flowering genes to control flowering [90]. Phytochromes exist in red (inactive) and far-red (active) absorbing forms, and the quality of the incident radiation (especially red: far-red) forms phytochrome photoequilibrium (PPE). Far-red LED has been used in plant applications to regulate flowering in at least some long-day plants. Red + far-red LED promotes flowering in long-day plants [91]. Adding far-red lighting from LEDs makes it possible to quick the flowering of some plants. Meijer et al. [70] reported that far-red light significantly accelerated flowering and increased the number of flowers per truss.

#### 2.1.4. Fruit Production

The fruit production of tomatoes in the LED and high-pressure sodium (HPS) treatments was insignificant, but it was higher than without lighting/natural light [74]. Even though there is no difference in results between HPS and LED treatments, the additional costs for LED applications are much cheaper than HPS lighting treatments [74]. However, treatment using LED lights can generally show better tomato performance than without lighting or HPS lighting treatment (Table 1). Red or blue lighting can increase the fruit numbers [83, 84]. A more significant fruit number or weight per plant will affect other plant parts (leaves and stems). Fruit is a strong sink, so more of the assimilate produced by the leaves (source) is distributed to the fruit. This is indicated by the higher fruit ratio per biomass, meaning the fruit's weight is higher than the other biomass (leaves and stems) [75]. Lighting at this wavelength stimulates the formation of chlorophyll and carotenoids; tomato plants do this to increase light absorption, thereby increasing the photosynthesis process [65]. Increasing the rate of photosynthesis means that it can increase the production of assimilate, which will be transferred to the sink, one of which is fruit, so that a more significant number of fruits can be produced, and their size is larger (Table 1). In tomatoes, far-red lighting increases fruit number, fruit weight, and yield [65–67]. In addition, previous research shows that red-blue lighting also increases fruit number, weight, and fruit ratio [74, 75, 94]. In addition, the glucose content and fructose content in the red and blue LED lighting treatments were higher than in other lighting treatments [64]. This is because red and blue lights are included in the optimum light spectra used in the photosynthesis process following light absorption in chlorophyll [64]. Soluble carbohydrates such as sucrose, glucose, and fructose are essential substrates that aid plant metabolism in various developmental and physiological events by regulating carbon transfer to metabolically active.

Artificial lighting also affects the color of tomatoes. The red color of tomatoes was influenced by the accumulation of lycopene [95]. Lycopene is a natural antioxidant in ripe tomatoes and is very important for human health [96]. Artificial lighting significantly impacts lycopene biosynthesis, incredibly blue light [80]. On the other hand, red lighting was involved in tomato carotenoid production through its receptor PHYs [97]. Applying blue or red lighting accelerates the color change of tomato fruit by stimulating lycopene synthesis through the regulation of downstream light signaling components, such as HY5 and PIF, which have the potential to control the expression of lycopene biosynthetic genes, such as phytoene synthase 1 (PSY1) encoding for tomato. PSY enzyme produced the first carotenoid, phytoene [8, 80]. The presence of red and blue lights activates PHY and CRY receptors, which play a role in absorbing light.

An overall overview of the effects of red and blue light spectra on photosynthesis, vegetative plant growth, flowering, and fruit yield in tomatoes is presented in Table 2.

### 2.2. LED Lighting Application in Postharvest Tomatoes

Tomatoes, one of the leading horticultural products, have a short shelf life and high losses. The leading causes of high loss at the postharvest level are weight loss, senescence, fruit softening, spoilage, and certain physiological disorders. Applying LED lighting during storage can be an alternative solution to reduce postharvest losses and maintain product quality because of its advantages in extending the shelf life of fruit and resulting in low heat emissions. Previous research showed that LED lighting treatment on tomatoes during storage increases shelf life, fruit hardness, and lycopene content (Table 3).

For selling tomatoes at short distances (nearby markets), tomatoes are needed whose skin color can quickly change to red without decreasing other qualities. This is because consumers tend to be more interested in red tomatoes. The lycopene content influences the red color of tomatoes. Red LED lighting treatment increased the lycopene content in tomato fruit [18]. Tomato fruit kept under red lighting at the ripe green stage increases lycopene accumulation, and this can be reversed after being exposed to far-red lighting, but fruit firmness and color are not significant [18, 106, 107, 112]. In addition, continuous red LED treatment on postharvest tomatoes also increased total phenolic content, total flavonoid, and antioxidant activity. However, firmness and chlorophyll content decreased, indicating that red lighting accelerates tomato fruit maturity [104]. In addition, red light treatment can also increase the soluble carbohydrates and anthocyanins and reduce the chlorophyll content in fruit [102, 113]. Anthocyanins are water-soluble pigments that are naturally found in various types of plants. Anthocyanins also play a role in regulating color changes in fruit. Carbohydrates in tomatoes are essential compounds. Appropriate LED lighting color spectrum with light absorption spectrum for plants can increase the production of assimilate stored in the sink (especially fruit). Furthermore, red LED lighting treatment also encourages the formation of lycopene and *β*-carotene in fruit, which causes the color of the fruit to change to red [102]. Light quality is an important environmental factor affecting pigment metabolism [114], and based on the results mentioned above, red light accelerates the breakdown of chlorophyll and carotenoid biosynthesis.

Furthermore, LED lighting with a higher red: far-red ratio in the postharvest phase induced lycopene synthesis in tomato fruit than other dark or light treatments, and LED lighting with a red: far-red ratio affected the titratable acidity and firmness of tomato fruit, depending on the cultivar [103]. Red: far-red lighting treatment reduces fruit firmness and increases the total acidity content [102, 103]. Red lighting encourages lycopene formation, which tends to be negatively correlated with fruit firmness.

Along with fruit ripening and coloring, the influence of light qualities on fruit flavor, such as sugar, acid, and aroma, is also interesting. Red-blue or far-red LED lighting increases the sugar content of tomato fruit [35, 93]. Treatment of tomatoes with red and blue light suppressed disease development and increased the production of carotenoids, TSS, and color without significant effects on firmness during postharvest storage. This suggests that postharvest lighting with red or blue LED lights regulates the synthesis and accumulation of carotenoids in tomatoes [108].

However, marketing tomatoes over long distances requires treatment that can extend the shelf life of tomatoes without reducing quality. Blue LED lighting treatment can slowly change the skin color of tomatoes to red and delay the decrease of tomato firmness [109]. Monochromatic blue light can extend tomatoes' shelf life by delaying fruit softening and ripening [109]. In contrast, additional blue light at 50 *μ*mol induced blue light receptor gene, CRY3 expression and promoted fruit lycopene synthesis and *β*-carotene accumulation in tomato fruit [80]. The transcription factor ELONGATED HYPOCOTYL 5 (HY5) mediates CRY-induced gene expression in response to blue light [115, 116]. Therefore, increased lycopene content can be regulated by increasing HY5 levels under supplemental blue light [80]. In addition, the contents of phytoene, *β*-carotene, *α*-carotene, and *γ*-carotene, as well as accelerated fruit coloration, are improved by the addition of blue light by increasing potassium uptake in the roots and transport in the fruit during ripening [117].

## 3. Conclusions

Climate change, global warming, and related environmental constraints can reduce agricultural land availability and production and change the postharvest handling of agricultural products. One of the commodities most affected by this is horticultural commodities. As one of the primary horticulture commodities, tomatoes are the most common crop produced in controlled environments with LED artificial lighting. Cultivation and postharvest of tomatoes can be optimized with LED artificial lighting. LEDs have several advantages over traditional light sources, such as emitting a narrow range of light, high purity and effectiveness, compact size, longer shelf life, and lower power consumption. In our review, we provide a comprehensive treatise on recent achievements in the use of LEDs for tomato cultivation and postharvest. The application of LED artificial light influences the cultivation and postharvest of tomatoes. Most light combinations (blue, red, red-blue, and far-red) have been studied extensively on the quality of tomato plants and fruit during cultivation and postharvest stages. Tomato leaves absorb the red-blue light spectrum to carry out photosynthesis, which is maximally induced by blue and red lights. In addition, red-blue LED lights increase the content of chlorophyll a, chlorophyll b, and carotenoids in tomato leaves and regulate vegetative growth in tomatoes. Tomato flowers are induced optimally with red + far-red LED lighting treatment. Red-blue lighting increases tomato production, such as fruit number, weight, and ratio. In postharvest tomatoes, blue LED lighting treatment can slowly change the color of the tomato skin to red, maintain firmness, and increase shelf life. Future research may be conducted on the effect of LED artificial lighting on the content of phytochemicals, antioxidants, and other essential nutrients in tomatoes. Different LED wavelengths can be explored to enhance various bioactive compounds and health-promoting components.

## Figures and Tables

**Table 1:** The mean value of fruit parameters of tomato on the light treatments.

Lighting treatment	Fruit number	Fruit weight (g)	Yield (g/plant)	Total carotenoid/lutein (*μ*g/g dry weight)	Lycopene (*μ*g/g dry weight)	Additional production cost per plan ($)	Reference
HPS	97	145.96	14,159			0.58	[74]
LED (RB)	94	142.61	13,406			0.15	
Control	67	135.32	9,067			—	
HPS	20	92.4	1,844	10.2	773.8		[92]
LED	22	96.7	2,101	24.7	910.3		
HPS			6,430				[93]
Red			9,090				
Red + far-red			9,680				
LED (FR: R)		37.7					[50]
Control		34.3					
FR-0	35.1	223		436.5			[65]
FR-8	37.3	229		466.7			
FR-16	36.4	228		521.3			
FR-24	36.6	228		476.6			

FR-0 = far-red 0 *μ*mol/m^2^s; FR-8 = far-red 8 *μ*mol/m^2^s; FR-16 = far-red 16 *μ*mol/m^2^s; FR-24 = far-red 24 *μ*mol/m^2^s.

**Table 2:** The impact of different LED light qualities on plant growth and development of tomato.

No	LED light wavelength	Photosynthetic photon flux density (PPFD), photoperiod, temperature, and relative humidity	Plant development	Reference
1	Red (664 nm), blue (446 nm), a mixture of red (60%) and blue (40%), and white (W)	12 h photoperiod, 190 ± 5 *μ*mol/m^2^s	R: stimulated hypocotyl elongation, cotyledon expansion, plant height, and leaf area, but produced seedlings with reduced photosynthetic capacity	[38]
			B: induced in seedlings the highest rubisco amount, more compact size, and reduced biomass	

2	Blue (457 nm) and red (657 nm)	(1) Monochromatic blue light (2) Monochromatic red light (3) Mixed RB light (3 : 1, RB: 75% of R light and 25% of B light). All of the treatments have been performed with a light intensity of 300 *μ*mol/m2s	R: photosynthesis in seedlings was inhibited by stomatal closure and caused a reduction in CO_2_ assimilation	[35]
			RB: influenced the photosynthetic pigment concentration, which increased the total chlorophyll	

3	Red and far-red	Low light intensity of 75 *μ*mol/m^2^s (LL) and with a relatively high light intensity of 300 *μ*mol/m^2^s	FR: reduced leaf maximum photosynthesis, leaf mass, thickness, and nitrogen, and increased the resistance to CO_2_ diffusion	[37]

4	Red-blue LED	Red: blue = 7 : 2, 12 h light period, with 300 *μ*mol/m^2^s of photosynthetically active radiation (PAR) at 26°C and 75% RH and 12 h dark period at 18°C and 75% RH	RB: increased the photosynthetic capacity and photosynthate production in tomato leaves	[44]

5	Red (650–670 nm) and blue (455–475 nm)	150 *μ*mol/m^2^s 16 h, 25 ± 1/20 ± 1°C, 65 ± 5%	RB: increased fresh and dry weight of shoots and leaf area, and displayed significantly higher rates of photosynthesis	[9]

6	Blue (454 nm) and red (663 nm)	A range of blue (B) light percentages: 0B, 25B, 50B, 75B, and 100B; the remaining percentage was red (R) light, with identical photosynthetic photon flux density (100 *μ*mol/m^2^s)	B: decreased plant height	[60]

7	Red (657 nm), blue (457 nm), purple (417 nm), white, and red: blue (1 : 1 and 3 : 1) combinations	12 h dark/light photoperiod and the same light intensity, which was a photosynthetic photon flux density (PPFD) of 300 ± 3 *μ*mol/m^2^s	R: increased glucose and fructose content	[64]
			R and RB: increased plant height and stem diameter	

8	Far-red (725–750 nm) LED	Intensity of 0, 8, 16, and 24 *μ*mol/m^2^s	FR: increased stem length, chlorophyll content, number and weight of fruit, and carotenoid content	[65]

9	Far-red LED	(i) 12 hours (FR 12) + 0.5–1.5 hours with very red LED	FR: increased stem length, stem internodes, weight of ripe fruit, leaf area, and fruit dry weight	[66]
		(ii) Irradiance was emitted from both sides of the lamps with a PPFD of 144 *μ*mol/m^2^s at 10 cm away from the lamps		
		(iii) Light intensity of the FR LEDs was 43 *μ*mol/m^2^s at 20 cm below the lamps		

10	White, white + far-red, far-red, red + blue, and red + blue + far-red	White, white +30 *μ*mol/m^2^s, red + blue, red + blue +30 *μ*mol/m^2^s FR, and RB +50 *μ*mol/m^2^s FR	FR: increased total plant biomass and tomato fruit production mainly by increasing dry mass partitioning of fruits	[67]

11	Far-red (730 nm)	Control (150 *μ*mol/m^2^s) and far-red (30 *μ*mol/m^2^s)	FR: increased plant height, accelerated flowering, and increased the number of flowers per truss	[70]

12	High-pressure sodium (HPS) lighting and red (627 nm) + blue (450 nm) LED	9 mol/m^2^ d; 95% red + 5% blue LED	RB: increased fruit weight and number of nodes (stem segments)	[74]

13	Red to blue	High intensity (135 *μ*mol/m^2^s), medium (115 *μ*mol/m^2^s), and low (100 *μ*mol/m^2^s), 16 hours (06.00–22.00), red to blue (5 : 1; 10 : 1, and 19 : 1), and the air temperature ranged from 21.8 ± 2.4°C during the day and 16.9 ± 3.4°C during the night	RB: improved fruit count, total fruit weight, fruit ratio, and biomass per plant	[75]

14	Red light (670 nm), blue light (455 nm), and red-blue light (1 : 1)	21 and 26°C, 300 ± 25 *μ*mol/m^2^s	RB: increased root growth and plant height	[76]

15	Blue (452 nm) and red (661 nm)	45 days in total (LED plants from 0 to 45 DAT and control plants from 30 to 45 DAT). Bidirectional LED lamps of 220 *μ*mol/m^2^s light intensity	RB: increased routine content in young leaves and mature leaves, flavonoids content in young leaf, and decreased flavonoids in mature leaves	[77]

16	Blue (430 nm) and red (660 nm)	12 hours (06.00–18.00), photosynthetic photon flux density (PPFD) was set at 50 *μ*mol/m^2^s and the illumination period was extended from 06: 00 to 18: 00 h every day, with ambient temperature and ambient humidity	(i) R and B: induced the synthesis of lycopene and *β*-carotene, and lutein induced earlier fruit maturing	[80]
			(ii) R: accelerated the red discoloration of the fruit skin	

17	HPS and red to far-red (FR: R) LED (450 and 660 nm)	18 h per day (04: 00–22: 00), 420 *μ*mol/m^2^s	FR: R LED increased the size of fruits	[50]

18	HPS, red/blue, and far-red	HPS (125 *μ*mol/m^2^s) + red/blue LED (106, 110, and 200 *μ*mol/m^2^s), far-red (200 *μ*mol/m^2^s), the duration of lighting to a maximum of 19 h	RB: increased fresh fruit weight, total dissolved solids, and sugar (glucose, sucrose, and fructose) content	[94]

19	Red (670 nm) and blue (435 nm)	LED supplemental lighting was applied from 06.00 h to 18.00 h	RB: increased stomatal and mesophyll conductance for photosynthetic CO_2_ uptake, supplemental lighting maximized photosynthetic productivity and consequently significantly increased water use efficiency, whether based on shoot dry biomass and fresh fruit yield, by 10.2% and 8.7%, respectively	[98]

20	Red-blue + far-red	(i) RB 150 *μ*mol/m^2^s + FR-0, 30, and 50 *μ*mol/m^2^s, photoperiod was set to 16 h	(i) FR: reduced weight loss, less pitting, faster red color development during shelf life (when prior long cold stored), and less softening	[99]
		(ii) Tomatoes stored in cold storage for 0, 5, 10, and 15 d at 4°C, followed by 20 d shelf life at 20°C	(ii) FR lighting during cultivation likely protects the membrane integrity and thus allows uninterrupted lycopene synthesis	

21	HPS, LED, and LED + IR	LED, HPS, and LED supplemented with an infrared (IR) heat source. Mean temperature 24.3 ± 0.03°C and mean relative humidity 62.7 ± 0.12%, 55 *μ*mol/m^2^s	LED + IR has been shown to boost the accumulation of bioactive chemicals, improve fruit quality, promote more rapid and early flowering in tomato plants, and can serve as an efficient replacement for traditional indoor illumination	[100]

22	Red (657 nm), blue (457 nm), and red-blue	RH 70%, 12 h/d photoperiod, 28/19°C day/night temperature and 400 *μ*mol/mol CO_2_ concentration, 300 *μ*mol/m^2^s	RB: enhanced the melatonin content in tomato fruit, which promoted the ripening of tomato fruit consequently and led to accelerated fruit softening as well as upregulation of ethylene and lycopene biosynthesis, respiration rate, antioxidant activity, and carbohydrate accumulation	[101]

**Table 3:** The postharvest effects of different LED lights on tomatoes.

No	LED light wavelength	Photosynthetic photon flux density (PPFD), photoperiod, temperature, and relative humidity	Postharvest effects	Reference
1	Red light (610–750 nm)	The tomatoes were exposed to all six red LEDs for 12 min, turned over, and exposed again for 12 min, equalling a total daily treatment energy of 24.3 KkJ/m^2^	R: increased lycopene in tomato exocarp	[18]
			R: as a regulator of carotenoid synthesis and accumulation	

2	Red light (665 nm)	113 *μ*mol/m^2^s, all tomato fruit were carefully turned over every day (at 2 pm) to ensure light exposition of both fruit sides	R: increased lycopene, ß-carotene, total flavonoids, and phenolics	[102]

3	(i) Red: far-red (R: FR) light ratio	64.05–92.60 *μ*mol/m^2^s, R: FR = 2.10–7.60	(i) R: FR ratio increased fruit firmness and titratable acidity	[103]
			(ii) R: FR ratio induced lycopene content	

4	Red light (665 nm)	113 *μ*mol/m^2^s, continuous red light for 10, 15, and 20 days	(i) R: increased lycopene, *β*-carotene, total phenolic content, total flavonoid concentration, and antioxidant activity	[104]
			(ii) R: positively influences metabolic processes and contributes to a higher content of health-promoting compounds	

5	Red light (665 nm)	113 *μ*mol/m^2^s, day and night temperatures (20.6 + 1.7 and 19.2 + 0.8°C) and day and night relative humidity (75.5 + 1.2 and 85 + 1.8%)	R: accelerated the color development and ripening and lycopene, *β*-carotene, total phenolic, and total flavonoid concentration	[105]

6	Red (638 nm) and blue (454 nm)	118 *μ*mol/m^2^s for 48 h and subsequently stored for up to 21 days at room temperature	(i) R or B: did not affect firmness and mass	[106]
			(ii) R: enhaced the color	
			(iii) R or B: increased lycopene, *β*-carotene, and TSS	

7	Blue (465 nm) and red (630 nm)	(i) Blue (B) intensity of 21.5 W/m^2^	(i) RB or B: increased the phytochemical biosynthesis (lycopene and naringenin, as main carotenoid and flavonoid found) compared to D (ii) RB or B enhanced the main bioactive compounds and carotenoids	[107]
		(ii) B and red (10.9 W/m^2^ and 11.3 W/m^2^)		
		(iii) Cold room at 5°C and a relative humidity of 85%		

8	Red (630 nm) and blue (450 nm)	160 ± 6 *μ*mol/m^2^s, all fruit were stored for 21 days at room temperature (21–23°C)	RB: suppressed the development of diseases, enhanced the production of carotenoids, TSS, as well as color, with no major effects on firmness during postharvest storage	[108]

9	Blue (440–450 nm) and red (650–660 nm) lights	(i) 85.72 *μ*mol/m^2^s and 102.70 *μ*mol/m^2^s	(i) B: slowed the color changes	[109]
		(ii) Storage in a dark room	(ii) B: proved to be effective in prolonging the shelf life of tomatoes by delaying fruit softening and ripening processes	

10	Blue light (430 ± 10 nm)	100 *μ*mol/m^2^s for 12 h per day (6: 00–18: 00)	B: the exposure to 30 min of blue light followed by an 8-minute pause resulted in the highest enhancement of lycopene, total phenolic compounds, total flavonoids, vitamin C, and soluble sugar in comparison to other treatments	[110]

11	Blue light (405 nm)	87 W/m^2^, temperature of 15°C and a relative humidity (RH) of 80%	B: potential to preserve the physicochemical quality of tomatoes and effectively control mold growth during transportation and storage	[111]

## Data Availability

The data used to support the findings of the study are included within the article.
